# Rare complications of infective endocarditis in marfan-like morphotype: diagnosis of multiple mitral valve aneurysms and aortic root abscess using three-dimensional transesophageal echocardiography

**DOI:** 10.1186/s12872-024-03715-3

**Published:** 2024-01-15

**Authors:** Meriem Boumaaz, Mohamed Reda Lahjouji, Raid Faraj, Najat Mouine, Iliyasse Asfalou, Aatif Benyass

**Affiliations:** 1 Department of Cardiology, Mohammed V Military Hospital, Mohammed V University, Rabat, Morocco; 2 Department of Cardiology B, Ibn Sina University Hospital, Mohammed V University, Rabat, Morocco

**Keywords:** Infective endocarditis, Marfan like morphotype, Mitral valve aneurysm, 3D-Transesophageal echocardiography, Bicuspid aortic valve, Cardiac surgery, Case report

## Abstract

Mitral valve aneurysm (MVA) is characterized by a saccular outpouching of the mitral leaflet, and it represents a rare condition typically associated with aortic valve endocarditis. Three-Dimensional Transesophageal Echocardiography (3D-TEE) serves as an effective tool for detecting the presence of MVA and its potential complications. In this report, we present a case involving a young man with striking images of bicuspid aortic valve endocarditis complicated by an aortic root abscess and multiple perforated mitral valve aneurysms, diagnosed using 3D TEE. This case suggests the uncommon coexistence of Marfan like morphotype, bicuspid aortic valve, and infective endocarditis as a triple mechanism in the occurrence of MVA. It underscores the significance of early and accurate imaging diagnosis for facilitating prompt surgical intervention.

## Background

Mitral valve aneurysms (MVAs) are rare complications, often arising in the context of aortic valve infective endocarditis (IE) and severe aortic regurgitation [1]. First reported in 1729 by Morand [2], recent studies show a low incidence of approximately 0.2–0.29% in patients undergoing transesophageal echocardiography (TEE) [3]. MVAs can lead to complications such as expansion, perforation, and significant valvular regurgitation. Timely diagnosis and surgical intervention are crucial to prevent these complications [4]. This case involves a young male with Marfan-like morphotype and bicuspid aortic valve (BAV) who developed aortic valve IE, multiple MVAs, and an aortic root abscess. Successful mitral and aortic valve replacement surgery, along with a six-week course of antibiotics, resulted in the patient's improvement. The case aims to enhance understanding of IE challenges in individuals with Marfan- like morphotype and contribute to medical knowledge, diagnostics, and treatment optimization for this specific population.

## Case presentation

**Fig. 1:**
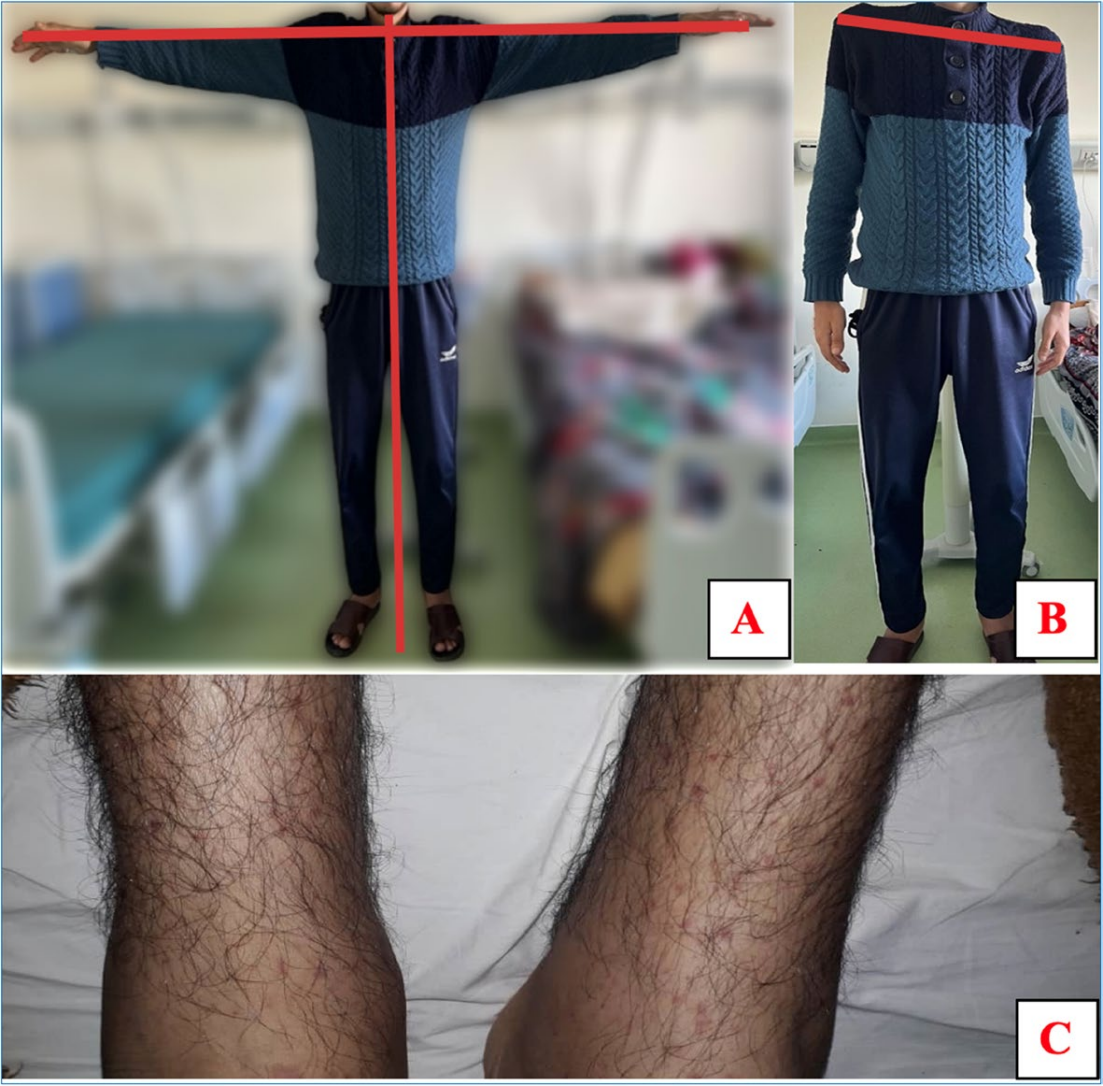
Patient’s morphotype **A** Tall and slender build with disproportionately long arms and excessive thinness **B** Inclination of the shoulders reflecting an abnormally curved spine **C** petechial purpura on patient’s legs

A 23-year-old man was referred to our Cardiology Center with a 3-month history of intermittent fever, night sweats, fatigue, and a productive cough with green phlegm. The patient reported no obvious risk factors for IE, and there was no cardiovascular disease in his clinical history. He has a known bicuspid aortic valve since childhood but had never undergone dental treatment or echocardiography follow-up. The patient exhibited phenotypic abnormalities suggestive of musculoskeletal impairment associated with Marfan syndrome, characterized by excess skeletal growth of the limbs, tall stature, large wingspan, excessive thinness, deformation of the trunk, and thin and long fingers. His height was 185 cm, weight 52 kg, with a BMI of 16.2 kg/m^2^ (Fig. 1-A, B). Additionally, he was followed for severe myopia.

**Fig. 2:**
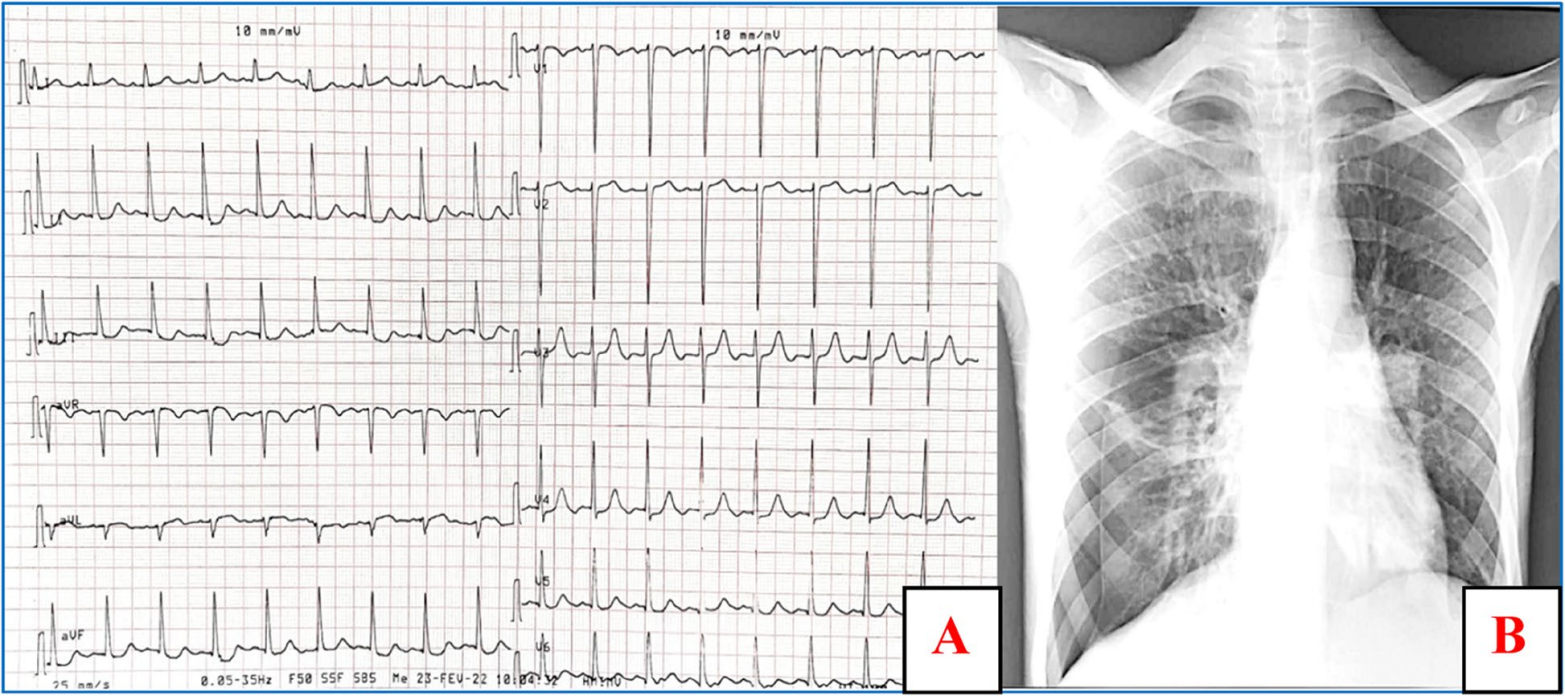
**A** ECG showing sinus tachycardia without atrioventricular and intraventricular conduction abnormalities. **B** chest x-ray showing bronchi dilation and pneumonia signs

Physical examination revealed a normal temperature and several dental cavities. Blood pressure was low (85/41 mmHg), pulse was 95 bpm, and there was petechial purpura on the lower extremities (Fig. 1-C). Cardiac auscultation disclosed a grade 5/6 diastolic murmur on the left sternal border and a grade 2/6 systolic murmur of mitral regurgitation at the apex. Oxygen saturation in ambient air was slightly reduced (SO2: 93%). The electrocardiogram (ECG) showed sinus rhythm with a heart rate of 95 beats/min and normal atrioventricular and intraventricular conduction; ventricular repolarization was substantially normal (Fig. 2-A). A chest radiograph showed signs of pulmonary bronchi dilation with perihilar and right lower lobe pneumonia (Fig. 2-B).

Blood analysis revealed a moderately elevated C-reactive protein level and white blood cell count with thrombocytopenia and hypochromic anemia. Blood cultures were positive for Streptococcus.

**Fig. 3:**
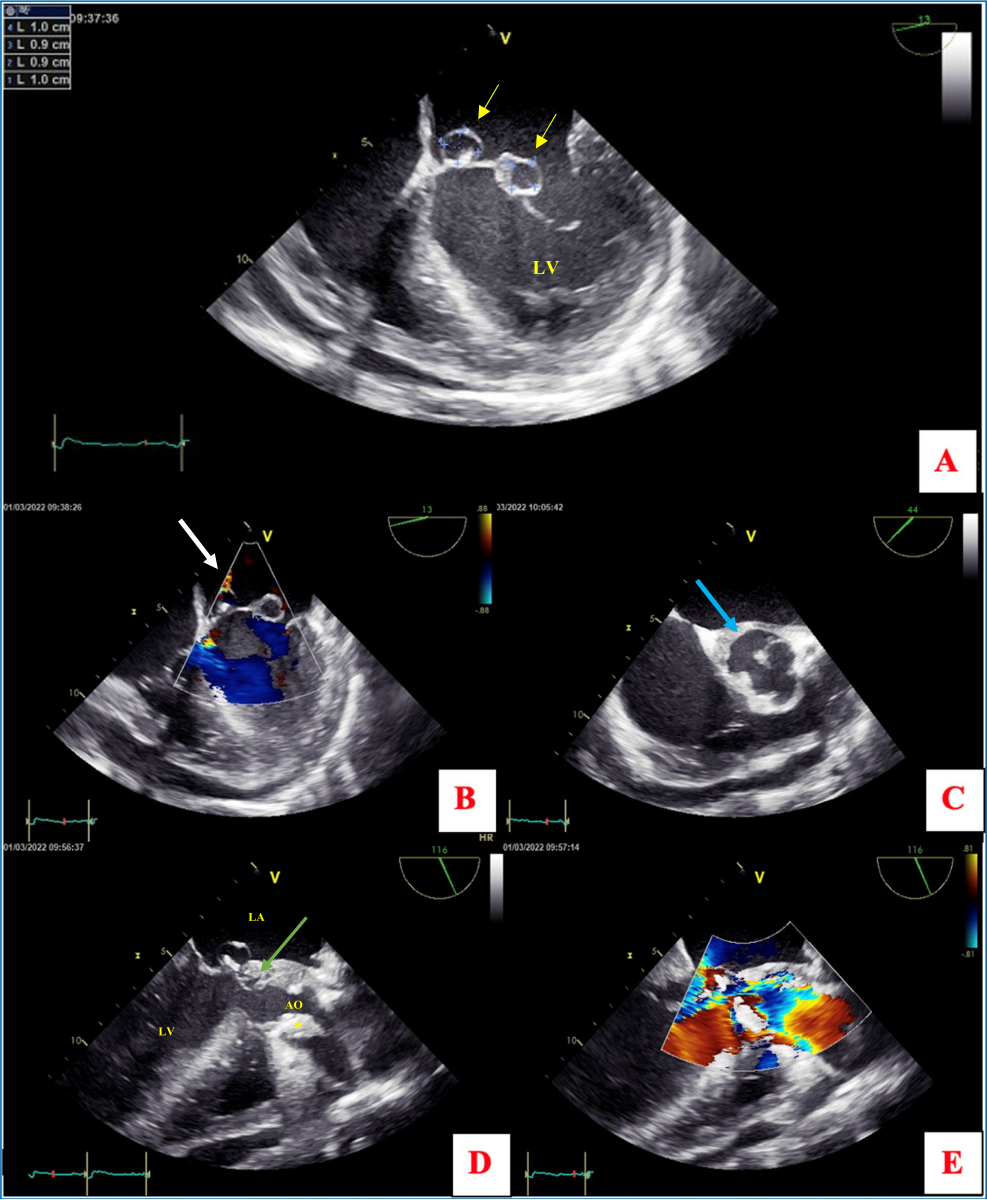
Transesophageal echocardiography (TEE) **A** Four-chamber mid-esophageal TEE showing two abnormal ring-like structures on the mitral valve compatible with aneurysms (yellow arrows). **B** Color Doppler analysis showing a turbulent flow through a small orifice within the medial anterior mitral valve leaflet aneurysm related to a mitral regurgitation (white arrow) **C** Mid-esophageal TEE short axis view showing a bicuspid aortic valve with thickening of the aortic-mitral fibrous trigone indicating an aortic root abscess (blue arrow) **D** three-chamber mid-esophageal TEE demonstrates an anterior mitral leaflet aneurysms juxtaposed to a prominent aortic root abscess (green arrow) and vegetation of the right coronary valve (*) **E** Color Doppler study showed a significant aortic regurgitation with an eccentric jet directed toward the anterior mitral leaflet aneurysm. LA = left atrium, Ao = ascending aorta, LV = left ventricle

**Fig. 4:**
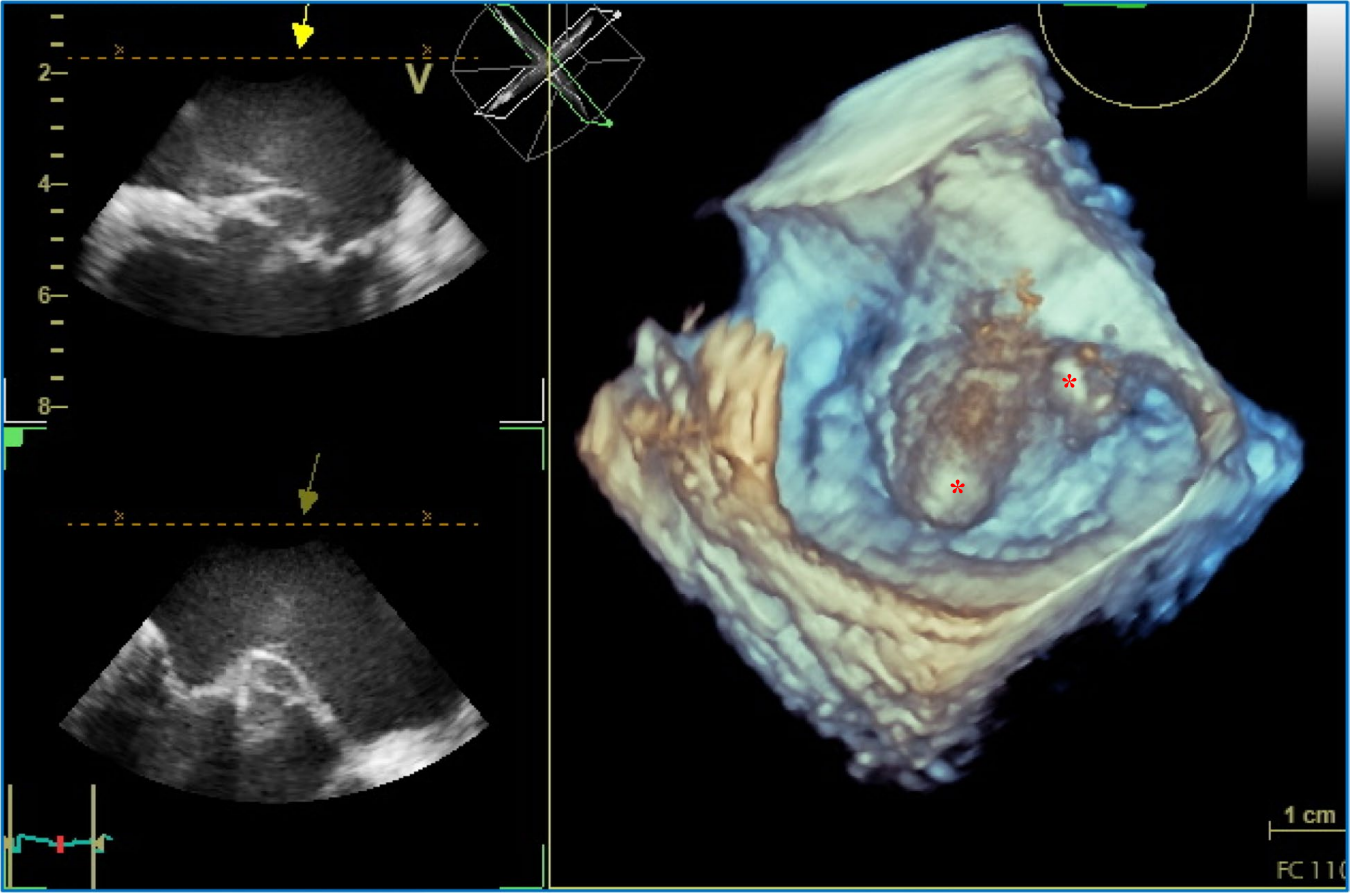
3D-TEE, observed from the left atrium towards the mitral valve annulus, showing two anterior mitral leaflet aneurysms protruding into the left atrium (*)

Transthoracic echocardiography revealed severe aortic regurgitation, a bileaflet aortic valve with a large vegetation on the right coronary cusps, mild mitral regurgitation, an enlarged left ventricle with an end-systolic diameter of 36 mm, end-diastolic diameter of 62 mm (36 mm/m^2^ indexed), normal contraction, an ejection fraction of 67%, medium abundance circumferential pericardial effusion, and pulmonary hypertension at 47 mmHg. The ascending aorta was dilated to 40 mm. Additionally, a lesion on the mitral valve was suspected. After stabilizing the patient’s hemodynamic condition, TEE was performed and confirmed these findings and revealed a bicuspid aortic valve (Sievers type 0), and an aortic root abscess measuring about 20 × 9 mm with a rollover movement of the right coronary cusp of the aortic valve resulting in eccentric severe aortic insufficiency and a torrential color jet that hit the anterior mitral valve leaflet (AMVL) (Fig. 3-C, D, E). TEE also showed two aneurysms on the atrial side of the AMVL, both measuring 10 × 9 mm, combined with mild mitral regurgitation through a small orifice within the aneurysm (Fig. 3-A, B), confirmed by 3D modality (Fig. 4). Computed Tomography scan extension assessment did not show septic embolization.

Due to the particular and rare situation characterized by an infection of the anterior mitral leaflet secondary to an infected regurgitant jet of a primary aortic IE in a bicuspid aortic valve, with an abscess and perforated aneurysm, the patient underwent prompt surgery for mitral and aortic valve replacement along with 6 weeks of antibiotic therapy.

Specimens collected during the surgical procedure were examined, and microbiologic findings confirmed the presence of Streptococcus pneumoniae.

Genetic testing to confirm Marfan syndrome was envisaged but was not possible due to financial constraints. He recovered uneventfully and was discharged asymptomatic on the 10th postoperative day. Additionally, all his dental caries were treated with antibiotic prophylaxis before the procedures. Three months later, the patient remained asymptomatic, and transthoracic echocardiography showed a perfect result of the performed treatment.

## Discussion

MVAs are infrequent but have the potential to be life-threatening. They predominantly occur on the AMVL, with occasional instances on the posterior mitral valve leaflet. The majority of MVAs are closely linked to IE. Non-infectious causes include severe mitral valve prolapse (MVP), congenital structural defects, and various connective tissue disorders like Ehlers-Danlos syndrome, Marfan syndrome, osteogenesis imperfecta, and pseudoxanthoma elasticum [5]. Additionally, LV outflow tract obstruction, hypertrophic cardiomyopathy, and the presence of bicuspid or quadricuspid aortic valves are reported as associated factors [4].

In our specific case, the weakening of the mitral leaflet resulted from the concurrent presence of Marfan like morphotype, BAV, and IE, ultimately leading to the occurrence of multiple MVAs. Notably, this particular observation has not been documented in the English-language medical literature, to the best of our knowledge.

Several proposed mechanisms elucidate the development of MVAs in the context of IE. These include: (a) the direct spread of infection through the mitral-aortic intervalvular fibrosa; (b) the impact of an infected aortic regurgitant jet on the anterior mitral leaflet, causing secondary infection and aneurysm formation (jet lesion); and (c) the direct contact of a prolapsing aortic vegetation [1].

In the case under consideration, a combination of the first two mechanisms is likely responsible for the occurrence of MVAs, as evidenced by a TEE study revealing an abscess in continuity with the mitral valve, and the aortic regurgitation from the infected BAV being directed towards the mitral valve and striking the anterior mitral leaflet.

TTE and TEE stand as the primary diagnostic imaging modalities [6], although 3D-TTE has proven highly valuable in this context as well. The echocardiographic manifestation of MVA is characterized by a saccular bulge towards the left atrium during systole, accompanied by diastolic collapse. During systole, blood flows into the saccular structure, while during diastole, blood flows out from it [7].

The differential diagnosis for MVA encompasses conditions such as MVP, flail mitral leaflet, chordal rupture, myxomatous degeneration of the mitral valve, papillary fibroelastoma, atrial myxoma involving the mitral valve, blood cyst of the papillary muscle, and anterior mitral valve diverticulum [3, 8].

Recently, real-time 3D-TEE has demonstrated the ability to present the spatial configuration of cardiac structures and their anomalies in a dynamic manner [9]. The superior effectiveness of 3D-TEE in elucidating the anatomical details of MVAs has been documented [4]. In this patient, 3D-TEE revealed multiple MVAs that were not detectable by TTE. Given that MVAs can lead to severe complications such as systemic embolization, leaflet perforation, and recurrent IE, their timely diagnosis and surgical intervention are of paramount importance [4].

The optimal management strategy for MVAs has not been definitively established. Some reports suggest that conservative management of uncomplicated MVAs with close follow-up may be feasible, depending on the degree of valve destruction and the anatomical disorder [10]. In instances where repair is deemed impractical [10], mitral valve replacement emerges as the sole feasible alternative [11]. Consideration for mitral valve surgery becomes imperative in scenarios involving MVA rupture, severe mitral regurgitation, or the necessity for aortic valve replacement surgery [12]. Timely surgical intervention for IE is generally recommended. Several case series have reported that early surgical treatment, as opposed to conventional approaches, results in superior long-term outcomes and a reduced risk of peripheral embolization [13], despite potential technical challenges associated with weakened and infected tissue. Administering a course of antibiotic therapy prior to surgery can help forestall potential extra-valvular involvement [14].

## Conclusion

MVAs are rare yet potentially life-threatening complications that necessitate careful consideration in evaluating patients with aortic valve endocarditis. They may result from the direct extension of infection to the mitral valve or significant aortic regurgitation with an eccentric jet directed toward the AMVL. TEE, especially the 3D-TEE, proves to be an excellent technique for confirming the diagnosis of an aneurysm and assessing the defect's severity. Valve replacement stands out as the most suitable treatment modality.

## Data Availability

No datasets were generated or analysed during the current study.
